# Peste Des Petits Ruminants Virus Infection of Small Ruminants: A Comprehensive Review

**DOI:** 10.3390/v6062287

**Published:** 2014-06-06

**Authors:** Naveen Kumar, Sunil Maherchandani, Sudhir Kumar Kashyap, Shoor Vir Singh, Shalini Sharma, Kundan Kumar Chaubey, Hinh Ly

**Affiliations:** 1 Virology Laboratory, Division of Animal Health, Central Institute for Research on Goats, Makhdoom, P.O. Farah, Mathura, UP 281122, India; E-Mails: shoorvir.singh@gmail.com (S.V.S.); kundan2006chaubey@gmail.com (K.K.C.); 2 Department of Veterinary Microbiology and Biotechnology, Rajasthan University of Veterinary and Animal Sciences, Bikaner, Rajasthan 334001, India; E-Mails: smchandani86@gmail.com (S.M.); skkashyap3@rediffmail.com (S.K.K.); 3 Department of Veterinary Physiology and Biochemistry, Lala Lajpat Rai University of Veterinary and Animal Sciences, Hisar, Haryana 125004, India; E-Mail: shalinisharma12_vet@yahoo.co.in; 4 Veterinary and Biomedical Sciences Department, University of Minnesota, 1988 Fitch Ave., Ste 295, Saint Paul, MN 55108, USA; E-Mail: hly@umn.edu

**Keywords:** Peste des petits ruminants, PPR, PPRV, vaccination, virus replication, disease resistance

## Abstract

Peste des petits ruminants (PPR) is caused by a Morbillivirus that belongs to the family *Paramyxoviridae*. PPR is an acute, highly contagious and fatal disease primarily affecting goats and sheep, whereas cattle undergo sub-clinical infection. With morbidity and mortality rates that can be as high as 90%, PPR is classified as an OIE (Office International des Epizooties)-listed disease. Considering the importance of sheep and goats in the livelihood of the poor and marginal farmers in Africa and South Asia, PPR is an important concern for food security and poverty alleviation. PPR virus (PPRV) and rinderpest virus (RPV) are closely related Morbilliviruses. Rinderpest has been globally eradicated by mass vaccination. Though a live attenuated vaccine is available against PPR for immunoprophylaxis, due to its instability in subtropical climate (thermo-sensitivity), unavailability of required doses and insufficient coverage (herd immunity), the disease control program has not been a great success. Further, emerging evidence of poor cross neutralization between vaccine strain and PPRV strains currently circulating in the field has raised concerns about the protective efficacy of the existing PPR vaccines. This review summarizes the recent advancement in PPRV replication, its pathogenesis, immune response to vaccine and disease control. Attempts have also been made to highlight the current trends in understanding the host susceptibility and resistance to PPR.

## 1. Introduction

Peste des petits ruminants (PPR) is a rinderpest-like disease of goats and sheep having many common names, such as ovine rinderpest, goat plague, plague of small ruminants or Kata. The French acronym PPR is commonly used worldwide. The disease was first described in 1942 by Gargadennec and Lalanne in the Ivory Coast, West Africa [1]. They identified the disease that was similar to but different from rinderpest in small ruminants which was not transmissible to cattle. In 1956, it was shown by Mornet and colleagues that the PPR virus (PPRV) and the rinderpest virus (RPV) are closely related antigenically (reviewed in reference [2]). In 1979, PPRV was classified as a Morbillivirus under the family *Paramyxoviridae* and the order *Mononegavirales* [3]. Currently there are seven known members of the genus Morbillivirus: measles virus (MV), RPV, PPRV, canine distemper virus (CDV), phocine distemper virus (PDV), cetacean morbillivirus (CeMV) and feline Morbillivirus (FMV) [4]. CeMV further grouped into three genetically distinct viruses, *i.e*. porpoise Morbillivirus (PMV), pilot whale Morbillivirus (PWMV) and dolphin Morbillivirus (DMV). PPRV was first isolated in sheep cell culture in 1962 [5] and was observed for the first time under electron microscope in 1967 [6]. 

The incubation period of the disease is 2–7 days. The disease is characterized by fever, oculo-nasal discharge, diarrhoea, leukopenia, dyspnea and sloughing of the epithelium of oral and nasal mucosa. The nasal and ocular discharge later becomes mucopurulent which gives fetid odor [7]. Pregnant animals may abort. Death usually occurs 4–6 days after the onset of fever. PPRV infection leads to high morbidity (up to 100%) and up to 90% mortality [8]. A close contact between the animals is essential to establish a successful infection of PPRV. Goats are usually more susceptible than sheep and the recovery rate is higher in sheep. Cattle can be infected with PPRV but is unable to transmit the disease to another host, though a sero-conversion against the PPRV H protein has been observed [9]. Antibodies against PPRV in cattle can provide cross protection against RPV [10]. Infection of cattle with PPRV also interferes with immune response against RPV [11]. PPR usually occurs year round though an association with season has been observed [12]. The clinical disease may be complicated by secondary infection with other pathogen such as those caused by *Pasteurella spp.*, *Escherichia coli* and *Mycoplasma spp.* [7]. Based on clinical signs, PPR may be confused with other diseases like capripox, bluetongue, contagious pustular dermatitis, foot-and-mouth disease (FMD) and contagious caprine pleuropneumonia [8]. Therefore, for differential diagnosis, confirmation must be performed by laboratory tests. 

## 2. Epidemiology

The disease is currently endemic in most of Africa, the Middle East, South Asia and China [13]. In the Indian subcontinent, the disease was reported for the first time in 1989 [14]. However, it was misdiagnosed for a long time as rinderpest and pasteurellosis until it could be well recognized [15]. As one of the largest sheep (71.5 million) and goat (140.5 million) rearing countries in the world, India considers PPR as one of the major and priority livestock disease [16]. The causative agent, the PPRV was first thought to be an aberrant strain of rinderpest virus that had lost its ability to infect cattle. Later it was shown to be antigenically and genetically distinct but closely related to RPV [3,17]. 

There are many gaps in current understanding about the epidemiology of PPR. There are many reports with different scenarios of animal species involved in the outbreaks: goats alone, sheep alone, or sheep and goats together. While large ruminants are believed to be relatively resistant, there have been reports indicating the involvement of PPRV in respiratory disease in camels [18] in Africa or rinderpest-like disease in buffaloes in India [19]. 

## 3. Economic Consequences

PPR is an OIE (Office International des Epizooties)-listed disease [20]. Sheep and particularly the goats (also known as poor man’s cow), contribute significantly to the nutrition and cash income of small farmers in Africa and South Asia, the two regions with the largest concentration (about 72.90%) of the poor peoples in the world [16,21]. The International Livestock Research Institute (ILRI), Nairobi, Kenya has identified PPR as one of the priority animal diseases whose control should be considered for poverty alleviation in Western Africa and South Asia, which highlights economic importance of PPR [16,22]. After the successful global rinderpest eradication program (GREP) in cattle, national and international organizations have started important initiatives to control PPR. In this regard, India started an ambitious program for PPR control in 2010. The United Nations Food and Agriculture Organization (FAO) and the OIE have also started two pilot projects in 2013 for the control of PPR in Africa with the financial support from the European commission and the Bill and Melinda Gates foundation, respectively. Very recently, countries in the SAARC (South Asian Association for Regional Cooperation) have prepared a roadmap for progressive control and eradication of the PPR in the region by 2020 [13].

According to the FAO estimates, the morbidity, mortality, production losses and treatment cost of PPR altogether are likely to cause an economic loss of $2,972.5 millions/year during 2012-2017 in SAARC region among which, in India alone, it would be $2569.00 million/year [13]. Global small ruminant population is about 1.8 billion, 62.5% (1.12 billion) of which is at risk for PPR [13]. If a 3-year mass vaccination policy is adopted, a total of 3.6 billion vaccine doses (1.2 × 3 = ~3.6) will be required at the cost of $3.6 billion (an average unit cost of a vaccine dose is $0.1 with the extrapolated unit cost of a vaccinated animal at $1.0). The capacity of vaccine manufacturing and fund raising for 3.6 billion doses of vaccine is a real concern. 

## 4. Virion Structure

PPRV virions are enveloped and pleomorphic in shape which varies in size from 150 to 700 nm [6]. The virions contain a negative-strand RNA genome enclosed in a ribonucleoprotein (RNP) core. The genomic RNA is packaged by nucleoprotein (N) to form nucleocapsid along with phosphoprotein (P) and large protein (L). 

### 4.1. Genome Organization

**Figure 1:**
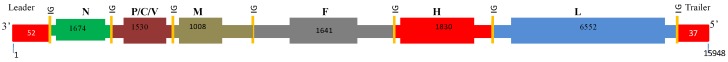
Genome organization of peste des petits ruminants virus (PPRV). Negative-stranded PPRV genome (RNA) containing 15,948 nucleotides and six genes that encode eight proteins. At the 3' and 5' ends there are untranslated regions (UTRs) of 52 nt and 37 nt, respectively. The terminator region of each gene is followed by a three nucleotide-long conserved (GAA) region called intergenic region (IG). IG region is also found at the junction of leader sequences and the N gene (first gene) and between the L gene (last gene) and the trailer region. Each transcription unit (to produce individual viral protein) is composed of the coding sequence(s), IG region and the conserved start and stop signals that flank the coding sequence(s). Besides the full length P protein, the open reading frame (ORF) of the P gene also produces two non structural proteins, namely C and V by alternative reading frame (leaky scanning) and RNA editing, respectively. IG = intergenic region, N = nucleocapsid protein, M = matrix protein, F = fusion protein, H = hemagglutinin protein, L = Polymerase (large) protein. Number indicates the length of nucleotides of the individual gene.

PPRV has a linear negative-stranded RNA genome that consists of 15,948 nucleotides and six genes that encode eight proteins (Figure 1). The 3' end of both genomic and antigenomic RNAs contains untranslated region (UTR), which serves as the promoter. At the 3' end of the PPRV genome, there is a leader region of 52 nucleotides. Similarly, at the 5' end of the genome there is a trailer region of 37 nucleotides. Fifty two nucleotide-long leader sequences together with the 3' UTR of the N gene and three nucleotide long intergenic regions (IG) between them serves as the genomic promoter (GP) for the synthesis of mRNA and complementary/antigenomic RNA. The antigenomic promoter (AGP) is composed of the trailer region and the 5'UTR following the stop codon of the L protein (the trailer region becomes the 3' end of the antigenomic RNA) and the IG region. The AGP only facilitates synthesis of the genomic RNA. A stretch of 23–31 nucleotides at the 3' terminus of both GP and AGP in PPRV is conserved and believed to act as an essential domain for the promoter activity [23]. Polymerase attaches at the 3' end and individual viral mRNAs are synthesized in the 3'-to-5' direction on the genomic RNA template. The terminator region of each gene is followed by a three nucleotide-long IG region (Figure 1). The IG region is also found at the junction of leader sequences and the N gene (first gene) and between the L gene (last gene) and the trailer region. The sequences of all the IG regions are conserved, *i.e*., GAA (CTT in mRNA). However, in the PPRV, at the junction of the L gene and the trailer region, GAA is substituted by GAU. At the junction of hemagglutinin (H) and L genes, some of the PPRV strains may contain GCA sequence. The IG region is preceded by a U-rich region that probably serves as polyadenylation signal. Each gene begins with the conserved UCCU/C sequence. Each transcriptional unit (to produce individual viral protein) is composed of the coding sequence, IG region and the conserved start and stop signals that flank it. These sequences together with sequences at 5' end of the next mRNA and sequences in the IG region are believed to regulate the stop-start mechanism of transcription. The sequences at the 3' end of the genome and IG regions are common features of Paramyxoviruses. At the start of each mRNA species in the PPRV and other paramyxoviral genomes, there is a conserved trinucleotide (AGG) sequence. Four conserved nucleotides (UUUU) are also present before each IG region of each mRNA transcript. Non-coding untranslated regions (UTR) varying in length are also present both before and after the open reading frame (ORF) of each gene. PPRV genome encodes six structural proteins namely the nucleocapsid protein (N), the phosphoprotein (P), the fusion protein (F), the matrix protein (M), the hemagglutinin-neuraminidase protein (HN), the large protein (L) and two non-structural proteins (C and V) in the order of 3'-N-P/C/V-M-F-HN-L-5' (Figure 1) [24,25,26].

#### 4.1.1. N Protein

Like other negative stranded RNA, PPRV genome is encapsidated by the N protein into ribonucleoprotein (RNP) complex. The molecular weight of the PPRV N protein is about 58 kDa [27]. N is a major viral protein produced in highest amount in Morbilliviruses [28]. Sequence similarities between Morbilliviruses N proteins range from 67% to 74% [29]. Expression of the N protein in bacterial, mammalian and insect cells leads to formation of nucleocapsid-like aggregates [30], which can be detected in both the cytoplasm and nucleus of transfected eukaryotic cells. The conserved region of the MV N protein that is responsible for self-assembly has been mapped and termed N_core_; only the wild-type but not mutated N protein can encapsidate the viral RNA [31]. 

#### 4.1.2. P Protein

P protein is also a component of the RNP and acts as a co-factor for the RNA-dependent RNA polymerase (RdRp). P protein is heavily phosphorylated at serine and threonine residues and interacts with both L and N proteins [29]. The predicted molecular weight of P protein is 60 kDa, however, in infected cell lysate it migrates at 79 kDa in SDS-PAGE partly due to heavy phosphorylation. The active P protein is a tetramer, oligomerization of which is required for efficient replication/transcription by the RdRp complex [32]. Among the Morbilliviruses, P protein is the least conserved protein [33]. The PPRV P protein is longest (509 amino acids) as compared to those of MV, RPV and CDV [34,35]. Serine residue at position 151, which is a potential phorphorylation site, is conserved among the Morbilliviruses [36]. The N-P interaction is not very stable which is probably required for the progression of the MV polymerase on the viral genome while it is copying the template RNA [37]. P protein has also been found to facilitate proper folding of the Sendai virus L protein [38].

#### 4.1.3. M Protein

M is a matrix protein of about 38 kD [39], which serves as a link between the N protein and the surface glycoproteins (H and F). It is the most conserved viral protein within the Morbilliviruses with an amino-acid identity level of up to 91% [40,41]. M protein of the Paramyxoviruses interacts with both envelope glycoproteins (H and F) and with the RNP complex in the cytoplasm [42,43]. By targeting the glycoproteins to the apical surface of the cells, mediated via tyrosine-dependent sorting signals located in the cytoplasmic tails [44], the M protein of MV plays an important role in the formation of progeny virus particles and their budding from the plasma membrane [45,46].

#### 4.1.4. F Protein

The F and H proteins form spikes on the surface of the viral envelope. Both play crucial roles during the initial steps of virus replication. The virus attaches to the membrane of the host via the H protein; then via the F protein, the viral and the cell membranes are fused, the process that allows the delivery of the viral nucleocapsid into the cell cytoplasm [47]. The predicted molecular weight of the F protein is 59.137 kDa. In contrast to other Morbilliviruses, which require both F and H/HN proteins for fusion, PPRV requires only the F protein [48]. The F protein in the Paramyxoviruses is synthesised as a precursor (F0), which is cleaved by the cytosolic enzymes into F1 and F2 fragments that are linked by a disulphide bond [49,50,51]. Cleavage of F0 in Morbilliviruses occurs at a conserved region of Arg-Arg-X1-X2-Arg (X1 may be any amino acid but X2 must be arginine or lysine), which is recognised by the Golgi-resident furin endopeptidase [52,53]. Cleavage of F0 is critically required for virus infectivity and pathogenesis, though not for virus assembly. The F1 fragment forms a membrane anchored subunit and has several conserved motifs, four of which are well known among the Paramyxoviruses viz: fusion peptide (FP), heptad repeat 1(HR1), heptad repeat 2 (HR 2) and a transmembrane domain (TM) [54,55,56,57,58,59]. During fusion of the viral and host cell membranes, FP is inserted into the host cell membrane and HR1 and HR2 interacts with each other to bring the viral and host cell membranes in a close proximity which in turn results in fusion [60,61]. Since these motifs in the F protein, which takes part in fusion, are common among the Paramyxoviruses, they may represent promising targets to develop antiviral therapeutics against a wide group of viruses [62,63,64,65].

The F protein of the Morbilliviruses is synthesized on the ribosomes of the rough endoplasmic reticulum (RER). During protein translocation, like all other membrane-associated proteins, the F protein of Morbilliviruses is also glycosylated by cellular enzymes at conserved N-linked glycosylation sites, Asn-X-Ser/Thr [66]. Beside fusogenic activity, glycosylation is believed to be essential for transporting proteins to the cell surface [67,68,69]. Morbillivirus F protein also has a leucine zipper motif, which controls the stabilization of the pre-fusogenic state and restrains the conformational switch, thereby preventing extensive cell-cell fusion activity [70]. Besides fusion, PPRV F protein also has hemolysin property; purified F protein causes lysis of the chicken red blood cells [71]. Both the F and H proteins of the Morbilliviruses have also been shown to induce autophagy [72].

#### 4.1.5. Hemagglutinin (Hemagglutinin-Neuraminidase) Protein

Unlike other Morbilliviruses, the H protein of the PPRV has both hemagglutinin and neuraminidase activities and hence is also named as Hemagglutinin-neuraminidase (HN) protein. The HN protein is responsible for virus attachment to host cell as well as cleavage of the sialic acid residue at the carbohydrate moiety in the glycoprotein of the host [73]. Morbillivirus H protein has a hydrophobic domain at the N-terminus (amino acid position 35–38), which remains associated in mature protein (not cleaved) and acts as a signal peptide to anchor the protein into the membrane [29]. Whereas N-terminal 34 amino acids of the Morbillivirus H protein are located inside the membrane, the C terminus is extruding outside, therefore defining it as a type II glycoprotein. Like the F protein, the H protein is synthesized on ribosomes of the rough endoplasmic reticulum (RER). As the protein progresses through the RER, modifications such as folding and oligomerization take place to form correct antigenic epitopes. After the ER, the protein passes through the Golgi-complex where it undergoes glycosylation. Degree of glycosylation contributes in determining the antigenicity and virulence of the virus. The numbers of N-glycosylation sites vary between different Morbilliviruses and between different strains of the same virus [29]. The predicted molecular weight of PPRV HN protein is 67 kDa, however, the recombinantly expressed PPRV H protein separates at about 70 kDa in SDS-PAGE [74].

Morbillivirus H protein is not very conserved among different strains. It has been found to down regulate CD46, a widely distributed cell membrane protein involved in the complement regulation on host cells [75]. Paramyxovirus glycoproteins vary in their ability to exhibit hemagglutinating properties [76]. Whereas PPRV and MV have been shown to agglutinate red blood cells [48,77], RPV does not [73]. 

#### 4.1.6. L Protein

The L protein (RdRp) with a predicted molecular weight of about 247.3 kDa is the largest viral protein and is expressed in the smallest amount in the infected cells. RPV and PPRV L proteins share 70.7% amino acid sequence similarity [24]. The L protein in combination with the P protein carries out replication, transcription, capping and polyadenylation of the viral mRNAs. The L protein of all Paramyxoviruses and several other negative-stranded RNA viruses contain three conserved domains, which perform various functions [78,79]. The first domain has conserved sequence KEXXRLXXKMXXKM from residues 1 to 606 and is believed to be an RNA binding motif. The second domain contains the conserved sequence GDDD flanked by hydrophobic regions (from residues 650 to 1694) and is believed to be the functional site for RdRp [29]. The third domain from residues 1717 to 2183 has kinase activity [29]. The L protein interacts with the P protein via its N‑terminal 408 amino-acid region at residues ILYPEVHLDSPIV, which is partially conserved among the Morbilliviruses [78,80].

#### 4.1.7. Nonstructural Proteins

Besides six structural proteins mentioned above, two non-structural proteins (C and V) are also produced from an alternative reading frame of the P protein of the Morbilliviruses. 

The C protein is about 20 kDa. It is translated from the P gene by an alternative reading frame at the second start codon. The C protein has high degree of homology within the Morbilliviruses [34]. Unlike the P protein, which is phosphorylated and localized only in the cytoplasm (with nucleocapsid), the C protein is not phosphorylated and localized either in cytoplasm and nucleus [81] or exclusively in the cytoplasm [82]. The C protein binds to the L protein and self interacts. The role of the C protein of the Morbilliviruses has been implicated in mediating efficient virus replication in peripheral blood cells [83], RNA synthesis [84], virulence determination [85], modulation of RdRp activity by interacting with the host cell protein SHCBP1 [86] and blocking induction of type I interferons [87]. 

The V is an another nonstructural protein produced from the P gene by a frame shift due to incorporation of a G residue at the conserved RNA editing site 5'-TTAAAAGGGCACAG-3'. The predicted molecular weight of V protein is 32.28 kDa. Unlike the C protein, the V protein is phosphorylated and can bind both N and L proteins and hence is believed to regulate RNA synthesis [82]. The V protein in the Paramyxoviruses has two evolutionarily distinct domains. The N-terminal domain, 75% of which is common among the viral P, V and W proteins, is not highly conserved between viruses, whereas the remaining 25% contain a cysteine-rich V-specific domain, which is conserved among all Paramyxoviruses. Each domain of the rinderpest virus V protein has been shown to perform distinct functions. The N-terminal domain binds STAT1, whereas the C-terminal V-specific domain interacts with the IFN receptor-associated kinases, Janus kinase 1 (JAK1) and Tyk2 and hence may be involved in blockade of IFN signaling [88]. Overexpression of the V protein leads to reduced RNA synthesis whereas its absence enhances the MV replication *in vitro* [89]. However, *in vivo* studies carried out on V-deleted MV exhibited reduced viral RNA synthesis and reduced virus load in mouse with consequently reduced pathogenicity [85].

Since non-structural proteins are quite conserved among the Morbilliviruses and many other Paramyxoviruses, they may play several functions during virus replication, virulence and evasion of immune responses. Therefore, additional studies are required to understand the precise molecular functions of these non-structural proteins.

## 5. Resistance to Physical and Chemical Action

The half-life of PPRV is about 2 hours (h) at 37 °C and it can be completely inactivated at 50 °C within 60 minutes. The virus is quite stable between pH 5.8–10.0 but is susceptible to the most common disinfectants such as alcohol, ether, phenol and sodium hydroxide [90]. The virus can survive for long periods in chilled and frozen tissues [90].

## 6. Virus Replication

### 6.1. Attachment and Entry

**Figure 2:**
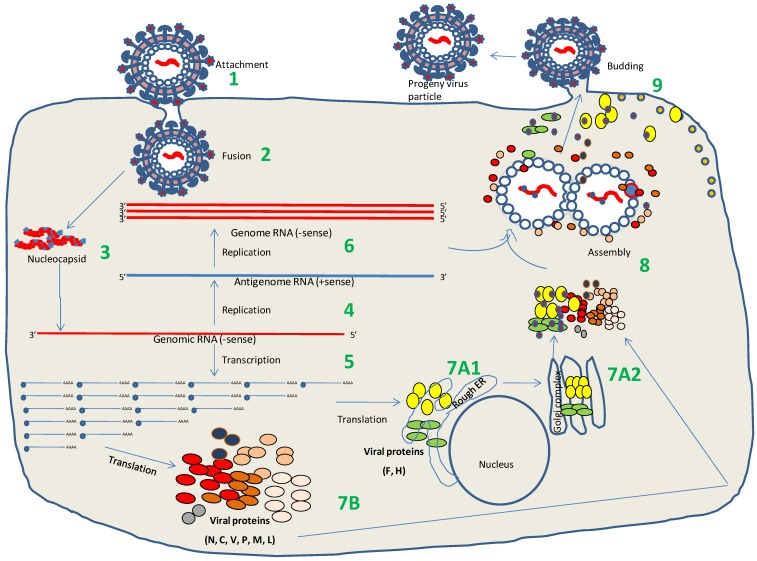
PPRV life cycle. (1). Attachment of the virus to host cell receptors (SLAM/Nectin-4) via its HN protein. (2). Fusion with plasma membrane via the F and HN proteins (3). Release of the viral genome into cytoplasm. (4). Genome replication by the virus-encoded RdRp of the RNPs (5). mRNA synthesis by the virus-encoded RdRp in the ‘start-stop’ mode (a mechanism of controlling the amount of individual protein being produced). (6). Synthesis of full-length positive sense RNA (antigenome RNA or complementary RNA, cRNA). (7). Synthesis of viral proteins: F and H proteins are synthesized on RER (7A1) and translocate across Golgi complex (7A2), where post-translational modifications take place. Other viral proteins (N, P, C, V, M, L) are synthesized on ribosomes (7B). (8). Assembly of progeny virions. (9). Budding of the progeny virions at the plasma membrane.

The life cycle of PPRV is 6–8 h in cultured cells [77]. The initial step of infection, *i.e.*, the binding of virus to the host cell and delivery of the nucleocapsid into host cell cytoplasm, certainly plays an important role in the pathogenesis of the virus and susceptibility to the host. The first interaction of the host and pathogen (attachment) is mediated by the binding of virus to the cell receptor (s) through its H protein (Figure 2). Similar to MV and CDV, PPRV has two natural cellular receptors: the signalling lymphocyte activation molecule (SLAM) or CD150 protein and Nectin-4. SLAM is exclusively expressed on immune cells (lymphocyte, macrophages and dendritic cell surface but not on epithelial cells), while Nectin-4 is the epithelial cell receptor, but is not expressed in lymphocytes and dendritic cells [91,92,93]. Existence of these two different receptors may explain why Morbilliviruses are both lymphotropic as well as epitheliotropic. The first receptor, SLAM, is certainly the most important, when the virus infects the host through the respiratory tract where it is taken by macrophages and dendritic cells and transported to local lymph nodes for multiplication. SLAM expression in peripheral blood mononuclear cells (PBMCs) is positively correlated with the magnitude of virus replication [91]. It has been shown that monkey cells expressing goat SLAM are more sensitive than those expressing cattle SLAM for PPRV isolation from pathological specimens [92]. The second cellular receptor, Nectin-4, has been found to play an important role for dissemination of MV throughout the body by facilitating amplification and subsequent release of the virus via different secretions (exit receptor) [94]. Region of the MV H protein that interacts with epithelial cell receptor has been mapped, and involves residues I456, L464, L482, P497, Y541 and Y543 [95,96]. However, some Morbilliviruses (CDV, MV) have been found to infect endothelial and neuronal cells, which neither express SLAM nor Nectin-4 (reviewed in reference [97]). Likewise, SLAM and Nectin-4 independent entry mechanisms has been observed in a variety of cell lines, though with reduced infectivity which suggests that alternative, ubiquitously expressed receptors also exist for some Morbilliviruses [98,99].

Paramyxoviruses enter to the host cell via fusion of viral and host cell membranes. During fusion, HR1 and HR2 domains of the F protein interact with each other to bring the viral and host cell membranes in a close proximity, which in turn results in fusion [60,61]. 

### 6.2. Transcription and Replication

Following release of nucleocapsid from the viral envelope, viral transcription starts in the cytoplasm. The RdRp present in the infecting virion initiates synthesis of the mRNA as well as complementary RNA (cRNA). The RdRp of all Paramyxoviruses is believed to attach at the genomic promoter (GP) on genomic RNA from where the transcription begins [24,29]. The individual transcriptional unit, which is composed of IG, coding sequences and conserved noncoding sequences flanking the coding region, is synthesized in the ‘start-stop’ mode. The RdRp can access the downstream transcriptional unit only after completion and release of the newly synthesized copy of the mRNA from the preceding unit (Figure 2). The RdRp may detach from the template during transcription at IG and may reinitiate the transcription at GP; a mechanism of controlling the amount of individual proteins being produced. The N protein, which is required in large amount, is located most closely to the GP and hence most abundantly transcribed (Figure 2). In contrast, the L protein is located farthest from the GP and hence transcribed in the lowest amount. Each mRNA species (different proteins) in the Paramyxoviruses is transcribed as naked RNA, which undergoes capping at their 5' end and polyadenylation at 3' end by the virus-encoded polymerase and hence is stable and can be efficiently translated by the host ribosomes [29]. Conserved trinucleotide (AGG) sequence present at the start of each mRNA species in the PPRV and other Paramyxovirus genomes is believed to be essential for proper processing of nascent mRNA and efficient gene expression, absence of which has resulted in premature termination of the transcript. Four nucleotides (UUUU) present before each IG region provide polyadenylation signals of the mRNA transcript [29].

Unlike five other genes, the P gene does not produce a single protein, rather it produces 3 different proteins (P, C and V). Besides synthesis of the full-length P protein from the first initiation codon, the C protein (a non-structural protein) is synthesised at the second AUG [29]. This leaky scanning of the ribosomes is probably due to the fact that the AUG is not located in perfect Kozak consensus sequences (A/GXXAUGG), which is required for efficient synthesis of the proteins [100]. The mRNA for another non-structural (C) protein is generated by co-translational editing by addition of one or more G residues in P mRNA at a conserved editing site (3'-AAUUUUUCCCGUGUC-5'). This insertion leads to a frame shift, from which a C-terminally truncated C protein is generated [101,102]. The requirement of the C protein for virus replication *in vitro* in human epithelial cells has been demonstrated in CDV [103]. Long UTRs, particularly present at the end of the M ORF and before the start of the F ORF in Morbilliviruses do not seem to play a major role in the virus life cycle as their deletion has very little effect on virus replication *in vitro* [104]. However, inclusion of UTRs has been found to increase the production of recombinant CDV proteins [105]. A recent study carried out on CDV has indicated that UTRs are required for virus virulence [106]. Non-coding RNAs are involved in various functions of Morbilliviruses, such as normal plaque formation [107], modulation of antiviral immunity [108,109] and establishing latent viral infection [110].

Some time after synthesis of the mRNA, the RdRp switches to synthesise full-length positive sense RNA (antigenome RNA or complementary RNA, cRNA). Like with the genomic RNA, cRNA is always associated with the N protein. It has been hypothesized that the accumulation of unassembled N protein in the cytoplasm plays a major role in switching the RdRp function from mRNA to cRNA synthesis [111]. Another model describes the existence of two different forms of RdRp, one for replication and another for transcription [112,113].

### 6.3. Virus Assembly and Release

The process of assembly and release of the Morbilliviruses including PPRV is not very well understood. Like other enveloped viruses, Paramyxoviruses also form virus particles when all the structural components of the virus, including viral glycoproteins and viral RNPs, have assembled at selected sites on the membranes where virions bud, then pinch off to achieve particle release (Figure 2), allowing the transmission of infections to new cells and hosts [114]. The viral M protein plays a crucial role in the assembly and release of Paramyxoviruses [115,116]. Highly abundant M protein serves as an adapter to link together the structural components of the virions (RNP cores and also with viral glycoproteins) and cellular membranes. Though M is the main protein that drives the Paramyxovirus assembly and release, other viral proteins such as H/HN, F and C also facilitate the assembly and budding process [114]. Incorporation of the genomic RNA into budding virions is driven by interactions between M and nucleocapsid at virus assembly sites [117]. Enveloped viruses may not encode all of the machinery required for efficient budding; instead, it may be supported by several host factors such as endosomal sorting complexes required for transport (ESCRT) and ubiquitin [114]. Paramyxovirus RNPs interact with the M protein under the plasma membrane (Figure 2) and buds via the ESCRT complex [118,119].

## 7. Immune Response

### 7.1. Innate Immune Response

The innate immune system can sense viruses, bacteria, parasites and fungi through the expression of pattern recognition receptors (PRRs), which recognize conserved structure in the pathogens called pathogen associated molecular patterns (PAMPs). The most common PRRs are toll-like receptors (TLRs), retinoic acid-inducible gene-I (RIG-I) like receptors (RLRs) such as RIG-I, melanoma differentiation antigen 5 (MDA-5) and Nod-like receptors (NLRs) [120]. The viral PAMPs such as dsRNA, uncapped ssRNA with 5' triphosphate, CpG DNA or specific viral proteins are sensed by at least three different types of PRRs viz: TLR3/7/8 [121,122], RIG-I/MDA-5 [123] and NLRP3 [124]. The engagement of the viral PAMPs with the PRRs leads to the activation of a complex network of intracellular signaling pathways, which ultimately results in transcription of several cytokine genes to produce an antiviral state in the host [125,126].

Interferons (IFNs) are the main group of cytokines that induce a virus-resistant state in host cells and also play an important role in modulating the adaptive immune response. The type I IFNs (IFNα/β) are produced as a direct response to virus infection and bind to the common IFNα/β receptor (IFNAR1/IFNAR2c) to activate transcription of interferon stimulated genes (ISGs) via JAKs (JAK1&JAK2)/STATs(STAT1&STAT2) pathway, which ultimately results in the establishment of an antiviral state in the cell. The type II IFN (IFNγ) is secreted by activated T cells and natural killer cells and mediates its functions through a different receptor (IFNGR). The IFNγ, upon binding to its receptor, induces formation of gamma activated factor (GAF) via JAKs(JAK1&Tyk2)/STAT1 pathways and activates transcription of a distinct subset of cellular genes that shape the IFNγ-mediated antiviral response [125,126].

PPRV infection in goats leads to a classic inflammatory response characterized by enhanced expression of cytokines such as IFNβ, IFNγ, IL-4, IL-1β, IL-8, IL-10, Il-6 and IL-12 [127,128]. However, Morbillivruses have been well known to inhibit IFN signaling. There have been some conflicting reports on Morbillivirus V protein’s ability to interfere with the IFN signaling [129,130,131,132,133,134,135,136]. However, in a recent study [137], while working simultaneously with RPV, MeV, PPRV and CDV, it has been observed that V protein of the Morbilliviruses block type I IFN action but has varying abilities to block type II IFN action because different morbillivirus V proteins have different abilities to interact (coprecipitate) with STAT1 and STAT2. 

Until now, there is very little information about the nature of PRRs triggered following PPRV infection. Further characterization of the innate immune receptors (PRRs) following vaccination/infection with PPRV will help identifying those PRRs that may play a significant role in generation of persistent antibody and cell-mediated immune response against PPRV.

### 7.2. Adaptive Immune Response

During Morbillivirus infection, severe immunosuppression is accompanied by a massive virus‑specific immune response. Protective cell-mediated and humoral immune responses against Morbilliviruses are directed mainly against H, F and N proteins [138,139,140]. Virus-specific antibody and CD4^+^ and CD8^+^ T cell responses have been observed following exposure to MV that contribute to virus clearance and protection from reinfection [141]. Envelope glycoproteins H and F of the RPV and PPRV induce a robust and protective neutralizing antibody response [142,143,144]. However, cell‑mediated immunity may play a role in protection. Though N is the most abundant viral protein, it does not induce neutralizing antibody response in the host [145]. However, it has been found to induce a strong cell-mediated immune response, which is believed to contribute to protection. In RPV and MV infection, N-specific T cells also constitute a major fraction of the virus-specific memory T cells [141,146,147]. Following a virulent PPRV infection in goats, the proportion of the circulating WC1^+^ γ/δ T-cells and CD14^+^ monocyte/ macrophage cells do not change. However, a decrease in the proportion of circulating CD4^+^ cells may be observed 4 days after challenge in naïve animals but not in vaccinated animals [148]. Also, there is a slight increase in the percentage of CD8^+^ T-cells at 7 days post-challenge in both naïve and vaccinated animals, suggesting induction of CTL responses by PPRV infection [148].

Attenuated Morbillivirus vaccines also induce cell-mediated immunity [149], which may be important for protection. It is not clear which immune effectors can be correlated with protection following vaccination with the PPRV vaccine: systemic neutralizing antibodies, cytotoxic T cell or mucosal immunity. Antibodies are most likely to be involved because passive transfer of immunity via colostrum may provide protection [150,151]. A study on rinderpest shows that induction of neutralizing antibodies by vaccination with purified viral proteins does not provide protection against infection [152], suggesting that it is not possible to infer protection based on antibody alone. Another study involving cattle recovered from RPV infection (even after more than 8 years and subsequently becoming almost antibody negative) shows that the animals resist challenge with a live virus, suggesting a strong anamnestic immune response exists [153]. It is assumed that the immunological memory in sheep and goats following PPRV vaccination is life-long. However, further experiments are required to test this assumption. 

Passively acquired maternal antibodies against PPRV in kids are usually detected up to 6 months with a gradual declining trend starting from the third month onwards. The protective titers are maintained until the fourth month. As maternal antibodies can interfere with vaccination, kids born from PPRV exposed or immunized goats must be immunized after 3–4 months [151,154].

## 8. Pathogenesis

The pathogenesis of PPRV is poorly understood, most of the knowledge is based on comparison with related Morbilliviruses, *i.e.*, CDV, RPV and MV. Though Morbilliviruses are known to induce immunosuppression, animals recovered from acute Morbillivirus infection usually develop a life-long immunity to reinfection [155], the molecular mechanism of which is not completely understood. During infection with PPRV, the virus is initially taken up by antigen presenting cells (APCs) present in the intraepithelial space and lamina propria of the respiratory mucosa (naso-pharyngeal/respiratory epithelium) [156,157] from where it is transported to regional lymphoid tissues where primary virus replication takes place. Then, via infected lymphocytes, infection spreads throughout the body via both the lymphatic and vascular systems [158,159,160]. PPRV is both lympho- and epithelio-tropic and infection usually results in conjunctivitis, rhinotracheitis, ulcerative stomatitis, gastroenteritis and pneumonia. Like other Morbilliviruses, PPRV is also cell associated; therefore it is thought that it reaches other organs/tissues by piggy bagging on the PBMCs [159]. The lung infection with PPRV is considered as a late event, occurring in the face of a high viral load [158].

PPRV leads to extensive necrosis in lymphoid organs (Peyer’s patches, spleen, thymus, pulmonary lymph nodes) and hence results in reduction (≥25%) in circulating peripheral blood leucocytes (leucopenia) [158,161,162]. The virus has also been found to induce apoptosis in PBMCs *in vitro* [163]. In a typical PPRV infection (experimental), fever usually develops within 3–7 dpi followed by onset of clinical signs which may vary depending on strain inoculated, route of administration and the immunological status of the infected animals. Clinical signs usually develop within 3–5 days following establishment of pyrexia. As disease progresses, mucosal hyperaemia, mucoid nasal discharges, anorexia and diarrhea are observed. Under natural infection, where dose of infecting virus is not as high as experimental infection, the incubation period is expected to be longer [161]. Upon exposure to virulent PPRV, susceptible animals usually develop acute pulmonary congestion and oedema and succumb to death within a week. Some of the animals in contrast, may develop a prolonged, chronic infection characterized by giant cell pneumonia, which may sometimes be complicated by bronchopneumonia. Such animals may survive beyond a month. The outcome of the infection depends on the ability of the animals to mount specific immune response to PPRV [164]. Factors such as co-infection with pre-existing parasitic organisms and nutritional status of the animal may also contribute in determining the disease severity and hence morbidity and mortality rates [165,166].

Histopathologically, PPRV also produces characteristic cytopathogenicity similar to other Morbilliviruses e.g., a large number of syncytia in the lymph nodes, splenic white pulp and gastrointestinal submucosal lymphoid tissue. The syncytia are followed by necrosis/apoptosis. Squamous epithelial syncytia are also observed in digestive tract epithelium and tonsillar and facial tissues [158]. Necrotic lesions in the intestinal lymph nodes probably lead to diarrhoea [155]. In contrast to the MV, RPV and PPRV are not associated with central nervous system complication. However, intracerebral but not intraperitoneal and intranasal inoculations of the RPV and PPRV may produce infection in mice [167]. A recent study also suggests that co-infection of the border disease virus (BDV) facilitates passage of the PPRV to the brain and hence results in infection of neuronal and glial cells [168].

To study the pathogenicity and to test the virulence of the PPRV strains, reliable and reproducible experimental challenge model for PPR in small ruminants have been developed [127,158,169,170,171,172] which may also be utilized to test vaccine efficacy. Using molecular and immunohistochemical methods to detect both viral antigens and nucleic acids, in an experimental PPRV infection that mimics natural infection, Pope *et al.* [158] has developed a clinical scoring system that enables disease symptoms to be graded and scored in order to enable ethical euthanasia of animals during late stage of the disease. 

The PPRV (nucleic acids) may be detected in oral and lachrymal secretions as early as 5 dpi, even before the appearance of visible clinical signs [158,173]. However, infectious virus cannot be demonstrated by virus isolation from conjunctival swabs until 7 dpi. Goats that recover from natural PPR outbreak shed the virus in the feces for about 12 weeks [174].

So far, only few studies have been carried out on PPR pathogenicity, events during virus propagation and its likely ways of replication in the host cell [175]. The reverse genetics approach developed of late [23,176,177] will likely provide important insights into the host range and molecular pathways involved in the pathogenesis and replication of PPRV.

## 9. Immunosuppression

The extensive damage of the lymphoid organs during Morbillivirus infection leads to immunosuppression. The level of infection in peripheral blood leucocyte and lymphoid tissues is directly correlated with immunosuppression. Immunosuppression may be caused by inhibition of IFNs production, altered cytokine response, suppression of the inflammatory response, direct infection and subsequent destruction of the leucocytes (leucopenia), inhibition of immunoglobulin synthesis (due to loss of B cells) and/or cell cycle arrest following direct contact with viral glycoproteins [178]. Though only a fraction of peripheral blood cells are infected, immunosuppression can last for weeks and hence increases the extent and severity of the pathological lesions [179]. The vaccine strain may also cause a transient immunosuppression [155]. With experiments carried out with MV and RPV, the importance of specific viral proteins (H, N and P) has been demonstrated to be responsible for inhibition of lymphoproliferation and induction of lymphodepletion [180,181,182,183,184]. The N protein of all Morbilliviruses binds to Fc-γ RII on human and murine B cells and inhibits *in vitro* antibody production [185] whereas the closely related Henipavirus also belonging to the *Paramyxoviridae* family fails to bind Fc-γ RII suggesting that Morbillivirus N and Fc-γ RII interaction is a common mechanism used to modulate immune response [185]. In addition, MV N protein has been found to inhibit T-cell proliferation, thus impairing the functions of dendritic cells and reducing inflammatory immune response in mice [186]. Immunosuppression is a multifaceted and complex process. Therefore, additional *in vivo* work is required to precisely understand the immunosuppression mechanism of Morbillivirus infections.

Extensive damage of the lymphoid tissues by Morbilliviruses may cause release of nucleic acids and proteins from the cells, and therefore, autoimmunity may also develop. MV infection has been found to be associated with several autoimmune disorders in human [155]. However, there is no documented evidence of autoimmune disease in goat/sheep infected with PPRV.

## 10. Host Susceptibility and Resistance to PPR

PPR is primarily a disease of small ruminants; both domestic and wild small ruminants are affected [187,188,189,190,191,192]. In general, goats are more severely affected than sheep, but some reports have highlighted cases of high mortality in sheep within mixed small ruminant flocks [14,193]. The seroprevalence of PPRV seems to differ notably among the species depending on the field conditions. According to some reports, in mixed populations, the serological prevalence rate has been found to be higher in sheep than in goats, which is attributed by the higher survival rate in sheep [8,194,195]. In contrast, some studies have reported that goats are more susceptible than sheep, and therefore have a higher probability of developing PPRV antibodies [196,197,198]. Interestingly, epidemiological surveillance studies carried out in different enzootic regions have revealed PPRV seroprevalence in other ruminants including cattle, buffalo and camel [194,195]. This seroprevalence can be as high as 41% and 67% in the case of buffaloes and cattle, respectively [9]. Cattle are considered, and probably buffaloes also, as potential dead-end hosts for PPRV. It appears however that this virus, for reasons not yet elucidated, can occasionally overcome the innate resistance of these species, resulting in the development of clinical signs. Nevertheless, development of clinical signs and case fatality has been observed experimentally in calves [199]. In India, a case fatality rate of 96% has been reported in domestic buffaloes (*Bubalus bubalis*) due to a virus that has been isolated and identified as PPRV [19]. Clinical and epidemiological investigations coupled with laboratory results have led to a strong suspicion of the role of PPRV in the emergence of an epizootic disease in dromedary populations in the Horn of Africa [18,200,201]. The viruses identified from sick dromedaries and small ruminants sharing the same grazing area have been found to be phylogenetically identical (reviewed in reference [202]). 

Like other RNA viruses, PPRV has the capacity to evolve rapidly due to its high replication rate and the relatively poor proofreading ability of the RdRp. Because of these characteristics, replication of these RNA viruses results in the generation of a unique viral population structure consisting of a large number of genetic micro-variants. In the case of viral pathogens, the presence of a variant from even at a minor proportion may have a profound clinical significance [203]. Unfortunately, such minor variants may not be detected by the traditional direct PCR-sequencing technology as it cannot capture virus variants that exist in less than 20%–25% of the heterogeneous virus population [203]. However, deep sequencing (Next Generation Sequencing, NGS) technology allows the detection of such variants even at level as low as 1% of the population [203]. With such a potential, NGS is an extremely powerful tool for investigating emergence/re-emergence of variants present in minor proportion and hidden in a viral population. In the case of PPR outbreaks in camels in Sudan, sequencing data of the virus has revealed similar sequence to that found in small ruminants that share the same grazing area [204]. These sequencing data were obtained by the classical direct sequencing of the PCR products. In addition, current PPRV molecular epidemiology data are based only on partial sequences of the genes encoding the nucleoprotein and fusion proteins [205,206]. For a study that aims to examine adaptation and co-evolution of PPRV, the first gene to be examined should certainly be the H protein, which is the attachment protein to the cell receptor and thereby is highly important in determination of the host range [180]. Since examining the structure of individual gene may not provide a comprehensive history of the organism due to the high rate of mutations, instead of analysing the unique H protein gene, NGS approach can be utilized for whole PPRV genome sequencing. Consequently, a genome-wide perspective is crucial to obtain a full understanding of evolutionary dynamics. For example, several critical mutations have been identified throughout the genome of an attenuated RPV; however, none of them is solely sufficient to completely attenuate the virus [207].

During *in vitro* replication of RPV and PPRV in lymphocytes, it has been observed that virulent RPV grows more readily in bovine than in caprine or ovine lymphocytes, whereas virulent PPRV grows better in lymphocytes from sheep and goats [208]. This characteristic feature might explain the difference in susceptibility/resistance of cattle and small ruminants *vis-a-vis* RPV and PPRV [15,209]. With those observations in mind and those related to the sensitivity of monkey cells expressing either goat SLAM or cattle SLAM in the isolation of PPRV, it can be postulated that one of the key elements in determining the differences between cattle and small ruminants resistance/susceptibility to RPV and PPRV may be linked to the interactions of the virus with host cell receptors SLAM and Nectin-4. Therefore, the mechanism of this interaction and the response of host cells (*in vitro* and *in vivo*) need to be explored in detail for better understanding of the pathogenesis of PPRV. 

To better understand the susceptibility/resistance to PPR, a new holistic approach that can be explored is systems biology. Unlike traditional biology, systems biology does not investigate individual genes or proteins one at a time, but it investigates the behaviour and relationships of all the elements in a particular biological system in a comprehensive, quantitative and integrative fashion (genome-wide gene expression profiling) [210]. Modern high throughput tools such as microarray, RNA seq, *in vitro* screening using siRNA and NGS have largely been used for global gene expression profiling. Systems biology may be useful in understanding differences in resistance/susceptibility to a particular disease in different animal species, identifying disease markers, identifying early markers of infection, prediction of the vaccine efficacy, identifying potential antiviral targets and understanding molecular mechanisms of the host-virus interactions [210,211].

## 11. Disease Control

The role of FAO and International Atomic Energy Agency (IAEA) laboratory in the control and eradication of rinderpest as part of FAO’s GREP has been appreciated worldwide. Due to significance from epidemiological and economic standpoints in many countries, PPR, subsequent to rinderpest, is one of the priorities for international organizations like FAO, IAEA, and OIE to control and finally eradicate it.

### 11.1. Vaccination

Vaccination is considered as the most effective way of controlling PPR. In the past, when a homologous vaccine against PPR was not available, a heterologous live attenuated tissue culture rinderpest vaccine (TCRP) (based on the antigenic similarity of PPRV with RPV) was used to control PPR [212]. The TCRP vaccine was shown to provide protection against PPR for about one year [10,213]. Later, the use of heterologous PPR vaccine was banned because it might have interfered with the GREP (to achieve status of rinderpest free zone) and to avoid handling of live RPV [11].

In order to develop a homologous PPR vaccine, adaptation of PPRV to tissue culture was first attempted in 1962 by Gilbert and Monnier when a cytopathic (CPE) effect of the virus was observed in primary sheep liver cells, manifested by the appearance of syncytia [5]. Later on, PPRV was adapted in Vero cells and a homologous live attenuated PPR vaccine was developed. The first one has been developed in Africa by continuous passage of Nigeria 75/1 strain in Vero cells and is now commonly used in African countries. Besides Africa, this vaccine was also used worldwide to control PPR in different endemic zones with different lineages of PPRV. Since lineage IV PPRV is restricted only in Asia, a lineage-specific (lineage IV) PPRV vaccine has been developed in India by continuous passage (N = 59) of Sungri/96 strain in Vero cells [214,215,216]. This vaccine is widely used throughout India. A sandwich ELISA (monoclonal antibody based) has been developed to rapidly perform virus quantitation of the vaccine and hence enhancing the quality testing of the PPRV vaccine [217].

A single dose of a PPR vaccine contains ~10^3^ TCID_50_ of Vero cell-attenuated PPRV and is believed to provide protective immunity in sheep and goats for about 4 years [8]. As the maternal antibodies can interfere with vaccination, kids born from PPRV exposed or immunized goats must be immunized after 3–4 months [151,154]. The vaccine is considered quite safe without any significant immunosuppressive effect on the host [218]. Animals vaccinated with an attenuated PPR vaccine are unable to transmit the challenge virus to animals with which they are in contact. Vaccination with one strain is believed to provide a complete protection for another strain of the same or different lineage of PPRV (reviewed in reference [8]), though a recently isolated strain (PPRV/Nanakpur) cross reacts poorly with monoclonal antibodies as well as the hyperimmune serum against Indian vaccine strain (Sungri/96) and hence raises concerns about cross protection among different PPRV strains [77]. The vaccine is thermo-sensitive; its shelf life is ~1 year at 4 °C [8]. The PPRV vaccine is mostly required for use in subtropical climate (South Asia and Africa). An effective cold chain is required to deliver the vaccine in the field under hot and humid climate, which is costly and inconvenient. Improved freeze-drying methods have increased the thermostabilty of the PPR vaccine that can resist temperature of 45 °C for 14 days without any major loss of potency [218]. Use of new stabilizers/diluents such as heavy water, trehalose, CaCl_2_, MgCl_2_, MgSO_4_ have significantly improved the thermostability of the PPR vaccines [219,220,221]. The PPR vaccine with improved freeze-dried method and stabilizers can be maintained at 37 °C for up to 24 h without any significant effect on the protective efficacy [221], although the reconstituted vaccine must be administered to the animals within 2 h. Further research is required to develop a thermo-resistant vaccine which would significantly enhance the success of the disease control program.

### 11.2. New Generation Vaccines

As per the OIE, in order to attain a disease-free status, the region (country) has to prove free from infection if the vaccine has been used for control and eradication of the disease. The current PPR vaccine has two major constraints: thermolability and inability to differentiate the infected from vaccinated animals (DIVA). 

Vaccinated animals produce high amount of neutralizing antibodies against the H, F and N proteins, similar to those recovered from a natural infection [28,222]. Current PPRV vaccines do not allow discrimination between infected-recovered animals from the vaccinated animals. A DIVA vaccine may be of great value in PPRV control and eradication campaign. PPRV has 6 genes, all of which are required for replication. Therefore, in order to create a DIVA version of the current live-attenuated PPRV vaccine, some additional proteins would be required to express in the viral genome. Alternatively, limited viral proteins may be expressed from an alternative virus vector, which may elicit immune protection without inducing the complete repertoire of antibodies that are induced following live-attenuated vaccination or with natural infection. 

Using vaccinia and capripox viruses as vaccine vectors, DIVA vaccines were developed for RPV [223,224] but could not be used as the GREP was in its final phase. Capripox virus vectors expressing PPRV glycoproteins have also been developed [225,226,227]. Besides being relatively thermotolerant, an additional benefit of using a recombinant capripox virus vaccine is that it would simultaneously vaccinate against two important pathogens (PPRV and goat/sheep pox) of small ruminants. However, recombinant capripox virus-vector based vaccine may not provide good antibody response because of pre-existing vector immunity and hence may not be an ideal candidate vaccine for DIVA. 

At the time of rinderpest eradication campaign, the areas that had been declared free of RP could not use the RPV vaccine strain to vaccinate against RP or PPR. Therefore, a chimeric RPV-PPRV recombinant virus (F and H gene swapped) vaccine was developed that protected goats upon challenge with the wild-type PPRV [228]. A chimeric RPV-PPRV marker vaccine (N protein) has also been developed that induces resistance against challenge with virulent RPV in cattle [229].

Modified vaccine virus Ankara (MVA) expressing PPRV F and H proteins has also been shown to induce resistance to challenge with virulent PPRV but two doses of vaccine must be injected prior to challenge [230], which is somewhat impractical for small ruminants. Fow-pox (FP) virus vectors, though proven successful as human vaccines, they elicit very poor antibody- and cell-mediated immune responses in ruminants [231].

At present time, a recombinant *Bombyx mori* nucleopolyhedrovirus displaying the immunodominant ectodomains of the F glycoprotein of PPRV and the H glycoprotein of RPV [232], the silk worm larvae-expressed recombinant F protein (reviewed in reference [220]) and the Semliki Forest virus (SFV) expressing the H protein [233] have all been developed. However, their efficacy has not yet been assessed in natural hosts (sheep/goats).

Replication-deficient adenovirus (Ad)-vectored vaccines induce potent antibody and cell-mediated (CD4^+^ and CD8^+^ T-cell) immune response [234]. Ad vectors also have an adjuvant effect [235], which may be further potentiated by co-expressing cytokines such as GMCSF, IL-2 and IL-12 [236]. Ad5 may be suitable for use in small ruminants since these animals have hardly any pre-existing immunity to the vector as has been observed in humans [237]. The thermostability of the Ad vectors can be improved [238,239] and its large-scale production is possible [240]. Vaccination of goats with Ad-H alone or in combination with an Ad-F have been found to induce potent antibody response [148,241,242], similar to those induced by live, attenuated PPRV vaccines [243] and induce resistance to challenge with virulent PPRV in goats [148]. Ad-H or Ad-H and Ad-F combination can also induce a potent effector memory CD8^+^ response in goats [148]. However, detailed mechanisms underlying protection induced by these vaccines are largely unknown. 

Considering the similar geographical distribution of diseases like PPR, sheep and goat pox, orf, some combined vaccines have been formulated without interfering with the immunogenicity of each other [227,244,245]. Besides reducing stress to the animals, the combined vaccines will be convenient and will cut cost of the overall vaccination package.

### 11.3. Progressive Control of PPR in SAARC Countries

FAO established an animal health regional support unit (RSU) in 2010 within the FAO sub-regional emergency centre for transboundary animal diseases (ECTAD) based in Kathmandu, Nepal for regional cooperative program on highly pathogenic and emerging diseases (HPED) in South Asia with a mandate to facilitate the coordination mechanism to harmonize disease control approaches and monitoring the progress of the implementation of activities in the SAARC countries. 

SAARC countries (India, Bangladesh, Pakistan, Afghanistan, Sri Lanka, Bhutan, Maldives and Nepal) have 40.5% of the global small ruminant population. PPR is one of the priority diseases in these regions. Except Sri Lanka, all SAARC countries are endemic to PPR. Considering the economic impact of PPR in livelihoods, RSU has taken an initiative to develop the 2011–2020 roadmap to control and contain PPR in the region and to strengthen the regional cooperative mechanism across borders. The FAO is likely to support countries in the region to implement the road map in preparing their national PPR control programs. However, capacity of the vaccine manufacturers and raising funds for over 3 billion doses of vaccine are real challenges. Therefore, targeted/strategic vaccination (immuno-sterilization) based on extensive epidemiological surveillance will not only reduce wastage of public funds but may also speed up disease eradication. Alternatively, in order to reduce the cost of the vaccination, rather than a 3-year mass vaccination strategy, mass vaccination in the first year followed by vaccination of young animals (newborns) only in the second year and a final mass vaccination in the third year may be adopted for effective control of the disease from the region [13]. The mass vaccinations of animals should be carried out a month before the expected seasonal movements, e.g., increased movements due to drought/dry spell, increased market activities-during religious festivals.

The delivery of the vaccine also increases the final cost of vaccination. Therefore, vaccinating against more than one pathogen that is prevalent in the area may further reduce the cost of the vaccine. In this regard, sheep and goat pox, contagious caprine pleuropneumonia (CCPP) and brucellosis, global distribution of which almost overlaps, are very suitable candidates for progressive control and possible eradication. 

Since different epidemiological situations may prevail in different countries, differential approaches of control and eradication need to be considered for countries that are: (i) free; (ii) free, but at high risk; and (iii) endemic. Animal movement across the international borders needs to be mapped in order to design an effective national risk-based strategic control plan. FAO has asked the member states to implement and annually update the national roadmap to identify indicators of progress toward PPR eradication by 2020. Availability of proper diagnostic test and quality-controlled vaccines that are in compliance with the OIE standards will be ensured by establishing a regional PPR vaccine bank. For monitoring the effectiveness of the program, post-vaccination protocols need to be developed and implemented at the country and regional levels. For diagnosis, the FAO has already established a SAARC regional laboratory for PPR in Bangladesh where member states can send their samples for confirmation of the outbreak. All the member states have been asked to create an immune belt by vaccinating along their international borders. 

### 11.4. Cross Protection within PPRV Strains

Based on partial sequence analysis of both the F and N genes, PPRV strains are grouped into four different lineages (I–IV) [220]. Although currently PPRV strains belong to all four lineages that are prevalent in Africa, all PPRV so far found in Asia are from lineage IV [220]. The Asian lineage (type IV) of PPRV was first reported in Africa in 2008 from the PPR outbreak in Morocco [204]. There is also a single report of lineage type III PPRV from Asia (India) [205]. Lineage classification may help in monitoring virus circulation and tracing the source of the outbreak as well as to prepare a homologous vaccine for adequate immunization. Moreover, it avoids risk of introduction of heterologous lineage hitherto not present.

Commercially available PPR vaccines (Nigeria75 or Sungri96) are believed to protect against all genetically defined lineages, which suggests there is no serological significance of lineage classification. Although the humoral responses from the vaccines tend to correlate with the level of protection from clinical disease, there might be a difference in the level of protection between homologous and heterologous challenge. The level of protection following vaccination may be influenced by the antigenic difference between the vaccine and field strains. Therefore the duration of immunity in sheep and goats following vaccination needs to be evaluated with homologous and heterologous PPRV strains. 

Recently, our laboratory has isolated a PPRV strain (PPRV/Nanakpur/2012) that poorly reacts with monoclonal antibodies as well as hyperimmune serum raised against the Indian vaccine strain (Sungri96). This virus cannot be captured in Antigen-ELISA (reagent based on the Indian vaccine strain), either. Moreover, PCR amplification of the PPRV H and F genes has been proven unsuccessful with at least 11 different pairs of primers, although these same primer pairs can amplify the genes of the vaccine strain. The virus can agglutinate chicken RBC in hemagglutination assay and exhibits CPE, characteristic of that of a Morbillivirus (fusion, syncytia, and degeneration). A 327 nucleotide-long fragment of the N gene, which is considered as quite conserved among different PPRV strains, has been successfully amplified by PCR and sequenced. The sequences are homologous to the PPRV strain (Accession Number DQ267191), which has convinced us that it is a PPRV. Our study suggests that some of the field PPRV strains may not cross-neutralized with antibodies against the vaccine strain and hence has raised concerns about the protective efficacy of the PPRV vaccine against heterologous strains [77]. The question is whether the cellular immune response generated against such PPRV strains can solely be responsible for providing cross protection. Serological and molecular characterization of the field PPRV strain is therefore of utmost importance.

### 11.5. Should Cattle Be Included under the Mass Vaccination Campaign for Progressive Control of PPR?

Rinderpest has been officially eradicated from the globe in 2011 [246]. PPRV and RPV belong to the same group of the Morbilliviruses with some immunological cross-reactions and relatively similar clinical signs. RPV causes an acute lethal disease in cattle, whereas sheep and goats develop subclinical infection. In contrast, PPRV causes an acute and highly fatal infection in goats but it doesn’t cause any clinical disease in cattle/buffalo (subclinical infection). During GREP, only cattle and buffaloes were the target species for mass vaccination but not sheep and goats [247]. Assumption has been made that PPRV emerged from RPV by natural passage (subclinical infection) in sheep and goats. Random sampling from cattle and buffaloes has shown seropositivity against PPRV suggesting evidence of PPRV infection in cattle and buffaloes [195]. At present time, there is a threat of emergence of virulent bovine PPRV in cattle. Therefore, the question is whether or not cattle should be included in the mass vaccination campaign against PPR, though it may not be economically viable to vaccinate cattle along with sheep and goats. 

### 11.6. Did Vaccination against Rinderpest Suppress the Upsurge of PPRV?

Since outbreaks of rinderpest in cattle transmitted the infection to nearby sheep and goats, assumptions have been made that the tissue culture-adapted RPV excreted from vaccinated cattle would have immunized the nearby sheep and goats, which might have suppressed the emergence of PPRV. Therefore, the upsurge of the PPRV has been considered to be as a result of the discontinuation of rinderpest vaccination following its eradication. However, laboratory experiment carried out with both RPV and PPRV suggests that only the virulent virus, but not live attenuated vaccine virus is able to self transmit from the infected animals to in-contact susceptible animals [248]. In this context, it would be interesting to develop a vaccine against PPR that self transmits the PPRV (albeit at lesser extent) to self immunize in-contact susceptible animals, including cattle. The current knowledge on PPRV suggests that, though it is able to infect cattle and some other animals in a way to make them seropositive, it has not yet succeeded to become bovine PPRV that can be maintained in nature without small ruminants.

### 11.7. Should PPR Be Eradicated or a Live-With Option Be Adopted?

The epidemiology and biology of the PPRV are very much similar to those of the RPV. Therefore, there are enough reasons to control and eradicate PPR very much in a similar way like rinderpest. Like RPV, there are several aspects that may favor eradication of PPR: (i) There is only one serotype of PPRV and it is believed that perfect cross protection appears to exist within strains from different lineages. (ii) Vaccine is considered to provide life-long immunity. (iii) There is no carrier state. (iv) A close contact between the animals is required for effective transmission of the disease. (v) Virus does not survive for a long period of time outside the host as it is readily destroyed by heat and sunlight and hence needs continuous source of susceptible animals for survival. (vi) Appropriate diagnostic tools are available. However, unless the vaccine is used sufficiently, widely and thoroughly to stop transmission of the virus in the endemic areas, it may simply be wasting the public funds and at worst helping the virus to perpetuate. The following parameters should be considered before implementing a progressive control program of PPR in order to eradicate it: (i) Mass vaccination to achieve a herd immunity (>80%) to block the effective transmission of the virus seems a difficult task in terms of the availability of the required doses of vaccines and veterinary infrastructure to cover all the animal population in rural cohorts. (ii) As mentioned under economic consequences of the PPR, the budget needed for a 3-year mass vaccination campaign to cover all the small ruminants population would be probably more than the actual economic loss caused by PPR. (iii) In consideration of the customs/cultural taboos prevalent in the countries endemic with PPR (South Asia and Africa), it seems difficult, particularly at the time of festivals, to restrict animal movement. (iv) The annual turnover rate of the small ruminants is much higher than the cattle, which means that herd immunity will be evaded more quickly. (v) Maintaining cold chain of the vaccine in rural areas is a difficult task. However, sharing cold chain facilities between human and animal vaccines under the one health program may cut the overall cost to facilitate the control program. (vi) The role of other ruminants (wild and domestic) in the maintenance of PPRV is not well understood. (vii) Though it is unlikely to happen with Morbilliviruses but few strains of PPRV may evade vaccine protection [77].

## 12. Antiviral Medication

There is currently no antiviral medication approved to treat sick animals suffering from viral infections in general and PPRV infection in particular. Antiseptic ointment can be applied to the sores of recovering animals and antibiotics should be administered to control secondary bacterial infection. Blocking spread of virus is of utmost importance to minimize the impact of viral diseases. Since vaccine cannot provide instantaneous protection, antiviral compounds could serve this purpose. Although limited *in vitro* and *in vivo* studies have been performed, encouraging results for FMD suggest that livestock could be protected against infection within 24 h following antiviral treatment and up to 12 h post-infection [249,250]. However, very few *in vitro* studies on the effect of potential antivirals against PPRV have been attempted [251,252] and currently there are no antiviral medications available to treat PPRV infection. 

Most antiviral agents so far approved (for human usage), are pathogen-specific and select for resistance because virus can mutate the druggable target. Infection of cells with viruses results in the activation of a variety of intracellular signaling pathways that in turn create an antiviral state. However, viruses have been known to exploit these signaling pathways to ensure efficient virus replication. This dependency by the virus on the host may be used to develop novel antiviral drugs [253,254]. Nuclear factor-kappa beta (NF-κB), Raf/MEK/ERK, receptor tyrosine kinase and phosphatidylinositol 3-kinase (PI3K) are important signaling pathways that are known to be required for efficient virus propagation and have attracted some attention as suitable targets for antiviral interventions [253,255,256,257,258,259]. These studies are in preclinical phase and will likely lead to a paradigm shift in antiviral drug development in terms of minimizing drug resistance because the virus cannot easily overcome cellular functions by simply mutating its genome. However, there is a significant gap in our understanding about how Morbillivirus interacts with the host cell signaling pathways, characterization of which may help in developing novel antiviral therapeutics agents. Though antiviral strategy may not be cost-effective for livestock, it could complement emergency vaccination or be applied to treat valuable zoological collections and breeding stocks. 

## 13. Conclusions

After the successful eradication of rinderpest from the globe, FAO has launched a progressive control program of PPR in the regions where it is currently present, by replicating the tools and experience used in the rinderpest eradication program. The vaccine against PPR is available, which is believed to provide protection at least for 3–4 years. However, a thermostable vaccine needs to be developed for a practical and effective vaccination. Moreover, emerging evidence suggests that some field strains do not cross-react well with the vaccine strain; therefore a close monitoring of the field strains is critical to ensure an effective vaccination program. PPRV causes an acute fatal disease in its natural host (sheep/goats), but a subclinical infection in catlle/buffaloes. There is a threat of the emergence of a new virulent Morbillivirus of bovine, similar in manner to the way PPRV has emerged in sheep and goats. Host susceptibility/resistance to PPRV is not well understood. A systems biology approach may be useful in unraveling the host factors associated with susceptibility/resistance to PPRV. In order to provide instantaneous protection and to avoid unnecessary preemptive culling during epidemics, anti-PPR therapeutics should also be developed.
